# Identification and validation of a novel prognostic signature based on mitochondria and oxidative stress related genes for glioblastoma

**DOI:** 10.1186/s12967-023-03970-6

**Published:** 2023-02-22

**Authors:** Shiao Tong, Minqi Xia, Yang Xu, Qian Sun, Liguo Ye, Fanen Yuan, Yixuan Wang, Jiayang Cai, Zhang Ye, Daofeng Tian

**Affiliations:** 1 Department of Neurosurgery, Renmin Hospital of Wuhan University, Wuhan, China; 2 Department of Endocrinology & Metabolism, Renmin Hospital of Wuhan University, Wuhan, China

**Keywords:** Glioblastoma, Prognosis, Risk score, Mitochondria, Oxidative stress

## Abstract

**Background:**

Mitochondria represent a major source of reactive oxygen species (ROS) in cells, and the direct increase in ROS content is the primary cause of oxidative stress, which plays an important role in tumor proliferation, invasion, angiogenesis, and treatment. However, the relationship between mitochondrial oxidative stress-related genes and glioblastoma (GBM) remains unclear. This study aimed to investigate the value of mitochondria and oxidative stress-related genes in the prognosis and therapeutic targets of GBM.

**Methods:**

We retrieved mitochondria and oxidative stress-related genes from several public databases. The LASSO regression and Cox analyses were utilized to build a risk model and the ROC curve was used to assess its performance. Then, we analyzed the correlation between the model and immunity and mutation. Furthermore, CCK8 and EdU assays were utilized to verify the proliferative capacity of GBM cells and flow cytometry was used to analyze apoptosis rates. Finally, the JC-1 assay and ATP levels were utilized to detect mitochondrial function, and the intracellular ROS levels were determined using MitoSOX and BODIPY 581/591 C11.

**Results:**

5 mitochondrial oxidative stress-related genes (CTSL, TXNRD2, NUDT1, STOX1, CYP2E1) were screened by differential expression analysis and Cox analysis and incorporated in a risk model which yielded a strong prediction accuracy (AUC value = 0.967). Furthermore, this model was strongly related to immune cell infiltration and mutation status and could identify potential targeted therapeutic drugs for GBM. Finally, we selected NUDT1 for further validation in vitro. The results showed that NUDT1 was elevated in GBM, and knockdown of NUDT1 inhibited the proliferation and induced apoptosis of GBM cells, while knockdown of NUDT1 damaged mitochondrial homeostasis and induced oxidative stress in GBM cells.

**Conclusion:**

Our study was the first to propose a prognostic model of mitochondria and oxidative stress-related genes, which provided potential therapeutic strategies for GBM patients.

**Supplementary Information:**

The online version contains supplementary material available at 10.1186/s12967-023-03970-6.

## Introduction

Glioblastoma (GBM) represents the most prevalent and aggressive kind of malignant diffuse glioma, accounting for over one-third of all brain tumors [1]. Comprehensive treatment consists of maximum safety resection combined with postoperative radiotherapy and chemotherapy with temozolomide (TMZ) [2, 3]. The median patient survival time is only 15 to 16 months, and the quality of life for late patients is extremely poor due to the high recurrence and drug resistance rates of GBM [4]. Therefore, GBM is a challenging medical problem requiring more in-depth and systematic mechanism research to improve treatment outcomes.

Mitochondria are the primary site of energy transformation and biosynthesis essential to the physiological functions of cells [5]. It is well-established that many diseases, including cancer, cardiovascular disease, and neurodegenerative diseases, are associated with mitochondrial dysfunction or injury [6]. Current evidence suggests that the mitochondrial reactive oxygen species (ROS) production by the mitochondrial respiratory chain is thought to be a key factor in tumor transformation, proliferation, and metastasis [7]. Low-level ROS can promote tumor proliferation, epithelial-mesenchymal transition (EMT) and angiogenesis in addition to taking part in signal transduction as a second signal. Mitochondrial dysfunction can lead to a sharp increase in ROS [8, 9]. Oxidative stress and possibly death can be induced once the antioxidant mechanism in cells is unable to eliminate the surge of ROS [10]. Anti-cancer therapy that targets the mitochondria may be a potential strategy. For instance, mitochondrial-related dynamin-related protein 1 (DRP1) regulated mitochondrial dysfunction and inhibited growth in pituitary adenoma [11]. Moreover, universal anti-cancer medications work directly by targeting the abnormal increase of ROS. Tai SH et al. found that the rise of ROS in glioma could be induced by TMZ [12]. However, the study on mitochondria and oxidative stress in GBM frequently focuses on a single factor and is not systematic or all-encompassing. Therefore, our comprehensive analysis of the prognosis and function of mitochondria and oxidative stress-related genes provides a novel and useful basis for treating and diagnosing GBM.

The past few years have witnessed significant progress in microarray technology and biological information analysis, which has resulted in more accurate and comprehensive molecular screening and targeted therapy [13, 14]. In this study, we aimed to build a prediction model for the prognosis and treatment of mitochondria and oxidative stress-related genes through bioinformatics. We obtained the mRNA transcription and clinical information of GBM patients from the TCGA database. At the same time, we retrieved mitochondria and oxidative stress-related genes. The least absolute shrinkage and selection operator (LASSO) regression analysis was conducted to construct a mitochondrial oxidative stress-related risk model. The model examined the immune response and treatment response in GBM, which exhibited high accuracy. Furthermore, additional in vitro experiments confirmed the model's reliability. In conclusion, our model identified potential diagnostic genes and provided novel treatment strategies for GBM patients.

## Data and methods

### Data sources

In this study, we collected three cohorts from public databases. Among them, mRNA expression data of GBM patients were obtained from the TCGA (https://portal.gdc.cancer.gov/) [15], GTEx (https://xenabrowser.net/datapages/) [16], and CGGA (http://www.cgga.org.cn/) databases [17, 18]. Clinical data obtained from TCGA and CGGA databases were shown in Additional file 3: Table S1. Finally, we used the "normalize between arrays" function of the "limma" package to reduce the effect of batch effects [19]. We downloaded a total of 2052 genes related to oxidative stress from MsigDB (http://www.gsea-msigdb.org/gsea/downloads.jsp) [20], GeneCards (https://www.genecards.org/) [21], and NCBI (https://www.ncbi.nlm.nih.gov/) databases [22]. 1387 genes related to mitochondrial stress were identified from the MsigDB database and Human MitoCarta 3.0 (https://www.broadinstitute.org/mitocarta/) database [23]. Finally, the c2. cp. kegg. V 7.5.1. symbols datasets were obtained from the MsigDB database.

### Construction of a risk score model

A combined cohort of TCGA and GTEx cohorts was used to screen for differentially expressed genes (DEGs) between GBM and normal brain samples. The "ggplot2" package was used to visualize the DEGs in two volcano plots, and the cutoff for DEGs was|LogFc|> 1 and P < 0.001. DEGs related to mitochondria and oxidative stress were intersected using the "VennDiagram" package. Additionally, the LASSO regression analysis was carried out using the "glmnet" package in order to avoid overfitting and the univariate Cox analysis was used to gain the prognostic-related genes (P < 0.05). Then, according to the regression coefficient and multivariate Cox analysis, genes and their regression coefficient were obtained to the most suitable λ value. Finally, we calculated the risk score of patients according to the algorithm: Risk Score = $\sum\nolimits_{{{\text{i}} = {1}}}^{n} {\text{coef(i)*}} x(i)$. Based on the median risk score (2.07658–2.07663), all TCGA and CGGA cohort samples were split into high- and low-risk groups. The survival analysis between the high- and low-risk groups were performed using the packages "survival" and "survminer," and the AUC value, which was used to assess the accuracy of the risk model, was calculated and visualized using the R package "survivalROC." Using the "survival" package, univariate and multivariate Cox analyses combining clinical data were carried out.

### Analysis of tumor immunity and mutation

The differences in immune cells and function between high- and low-risk groups were compared using single sample gene set enrichment analysis (ssGSEA) and an estimation algorithm. The "ESTIMATE", "ggpubr," and "limma" packages were then used to compare variations in tumor microenvironments between the high- and low-risk groups. Data on 168 GBM patients' somatic mutations were downloaded from TCGA. The number of insertion/deletion and replacement mutations per million bases is known as the tumor mutation burden (TMB). The "maftools" package was used to analyze the mutation in the high- and low-risk groups. Based on the median TMB score, all samples were divided into high and low TMB groups. The difference in survival between high and low TMB groups was examined by Kaplan–Meier survival analysis.

### Analysis of functional enrichment

Using the package "clusterProfile", we conducted Gene Ontology (GO), Kyoto Encyclopedia of Genes and Genome (KEGG), and Gene Set Enrichment (GSEA) enrichment analysis. P < 0.05 and an FDR < 0.25 were regarded as statistically significant. The GSEA software version was 4.3.2, and we utilized the c2. Cp. Kegg. 7.5.1 dataset, the minimum gene set was set to 15, the maximum gene set to 500, and the samples were resampled 1000 times.

### Drug sensitivity

To assess the relationship between drug sensitivity and risk score and the differences between high- and low-risk groups in drug sensitivity, data on drug sensitivity and CCLE expression were obtained from the PRISM dataset (https://depmap.org/repurposing) [24].

### Immunohistochemistry (IHC)

IHC was utilized to analyze the protein expression level in tumor tissues. First, the tissue sections were deparaffinized and rehydrated. A citrate buffer was used for antigen retrieval at 95 °C for 10 min and 0.3% hydrogen peroxide was utilized to remove endogenous peroxidase for 20 min. Then, the sections were blocked with 5% normal fetal bovine serum (FBS) at room temperature for 40 min and reacted with the corresponding anti-NUDT1 antibody (ZENBIO, 389021, 1:100) and secondary antibody (Servicebio, G1213, 1:100). Finally, representative IHC pictures were obtained using optical microscopy (Olympus, Japan) after positive signals were marked with DAB solution and nuclei were visualized with hematoxylin.

### Immunofluorescence

The cells were immobilized by paraformaldehyde for 15 min, and 0.2% Triton X-100 was utilized to rupture the cell membrane. The cells were exposed to the relevant primary antibody at 4 °C after being blocked by 5% FBS. On the second day, the cells were treated with HRP-conjugated secondary antibody at room temperature for 25 min. Finally, the fluorescence pictures were taken using confocal microscopy (Olympus FV1200, Japan).

### Cell culture and transfection

All GBM cells in this article were obtained from the Tumor Cell Repository of the National Cancer Institute (Frederick, USA). These cells were grown in the optimal conditions of 37 °C, and 5% CO2 in high-Glucose media supplemented with 15% FBS (Hyclone, Australia) and 1% penicillin–streptomycin solution (Biosharp, China). According to the manufacturer's instructions, the Lipofectamine 3000 reagent (ThermoFisher, USA) was employed to transfect 2 ug of the NUDT1 plasmid (GenePharma, China) into cells when the cell density reached roughly 85%. The plasmid sequence of NUDT1 included the forward sequence (GGUUCCAGCUGGAUCAGAUTT) and the reverse sequence (AUCUGAUCCAGCUGGAACCTT).

### Detection of cell proliferation

For the CCK8 assay, 5000 transfected cells were planted and grown in 96-well plates. When the cells were cultivated for 24, 48 and 72 h, respectively, 10 μ CCK8 reagent (Biosharp, China) was added to the corresponding wells and reacted with the cells for 1 h. Finally, a microplate Reader (SpectraMax ABS, China) was utilized to analyze and record the absorbance at the 450 nm of these cells.

For the colony formation assay, 500 transfected cells were planted and cultured in 6-well plates. After 14 days, the colonies were recorded and counted after the cells were fixed and stained.

For the EdU assay, the transfected cells were immobilized for 15 min with paraformaldehyde after responding for 2 h to the EdU solution (Solarbio, China) of 50 μM at 37 °C, and the DAPI reagent (Sigma, USA) was employed to color the nucleus for 10 min. The representative images of EdU were then captured using a fluorescent microscope (Olympus BX53, Japan), and the EdU positive cells ratio was evaluated as the ratio of red versus blue fluorescent cells.

### Detection of cell death

For the cytotoxicity assay, the transfected cells were reacted with Calcein AM/PI solution (Beyotime, China) for 40 min in the dark. After these cells were washed with phosphate buffered saline (PBS) 3 times, the fluorescent microscope (Olympus BX53, Japan) was utilized to observe and count the live/dead cells. The living cells were labeled with Calcein AM and displayed green fluorescence, while the dead cells were labeled with PI and displayed in red fluorescence.

For apoptosis assay, the transfected cells were collected by centrifugation and lightly washed with PBS 2 times. Then, the 5 μl Annexin V-PE/7-ADD kit (Becton, USA) was utilized to incubate the cells for 15 min and the apoptosis rate was analyzed and recorded by a flow cytometer (Beckman, Japan).

### Measurement of mitochondrial membrane potential (MMP)

JC-1 reagent (Beyotime, China) was utilized to detect the MMP of GBM cells. According to the instructions of the manufacturer, the transfected cells reacted with JC-1 solution at 37 °C for 30 min, and DAPI solution (Sigma, USA) was employed to color the nucleus. Finally, JC-1 fluorescence variations were monitored and recorded using a fluorescence microscope (Olympus BX53, Japan). JC-1 aggregates (red fluorescence) changed into JC-1 monomers (green fluorescence) as the MMP decreased.

### Transmission electron microscope (TEM)

TEM was utilized to detect the subcellular structure of mitochondria. The transfected cells were gathered and immobilized using 2.5% glutaraldehyde at 4 ℃. The next day, uranyl acetate and lead acetate were utilized to visualize cells after dehydration using an ethanol gradient. Finally, an electron microscope (JEM-1400Plus, Japan) was used to observe and photograph the cells.

### Western blotting (WB)

Intracellular proteins were extracted through RIPA lysate (Beyotime, China) and gradient separated using 10% sodium dodecyl sulfate–polyacrylamide gel electrophoresis (SDS-PAGE). These proteins were then applied to PVDF bands and reacted with anti-tubulin (Abmart, M20005, 1:2000), anti-NUDT1 (ZENBIO, 389021, 1:100), anti-DRP1 (Proteintech, 12957-1-AP, 1:1000) and anti-MFN2 (Proteintech, 12186-1-AP, 1:1000) antibodies at 4 °C. The next day, these bands were reacted with HRP-labeled secondary antibodies (Servicebio, China) at room temperature for 30 min and visualized using enhanced ECL mixed solution (Biosharp, China).

### Measurement of ROS production

For mitochondrial ROS, 5 μM MitoSOX solution (Invitrogen, USA) was utilized to react with transfected cells for 15 min. And the 100 nM Mito-Tracker probe (Beyotime, China) was utilized to locate mitochondria.

For intracellular total ROS, 5 μM Dichlorofluorescein diacetate (DCFH-DA) solution (GOYOO, China) was used to incubate with GBM cells for 30 min at dark to detect the total ROS. After these cells were cleaned with PBS 2 times, the DCF fluorescence was detected by a flow cytometer (Beckman, Japan) and ImageJ software was utilized to visualize the results.

For intracellular lipid ROS quantification, the 2 μM BODIPY 581/591 C11 dye (Thermo Fisher, USA) was used to react with transfected cells for 45 min to assess the lipid ROS and DAPI solution (Sigma, USA) was performed to stain the nucleus. After these cells were washed with PBS 2 times, the BODIPY image was recorded by a fluorescence microscope (Olympus BX53, Japan).

### Statistical analysis

All data were analyzed by R studio (version 4.2.1) and GraphPad Prism (version 9.0.0) in this study. Student's t-test was utilized to analyze the difference between two groups if the date was normally distributed, otherwise, Mann–Whitney U test was utilized. P values less than 0.05 were statistically significant.

## Results

### Study flowchart

**Fig. 1:**
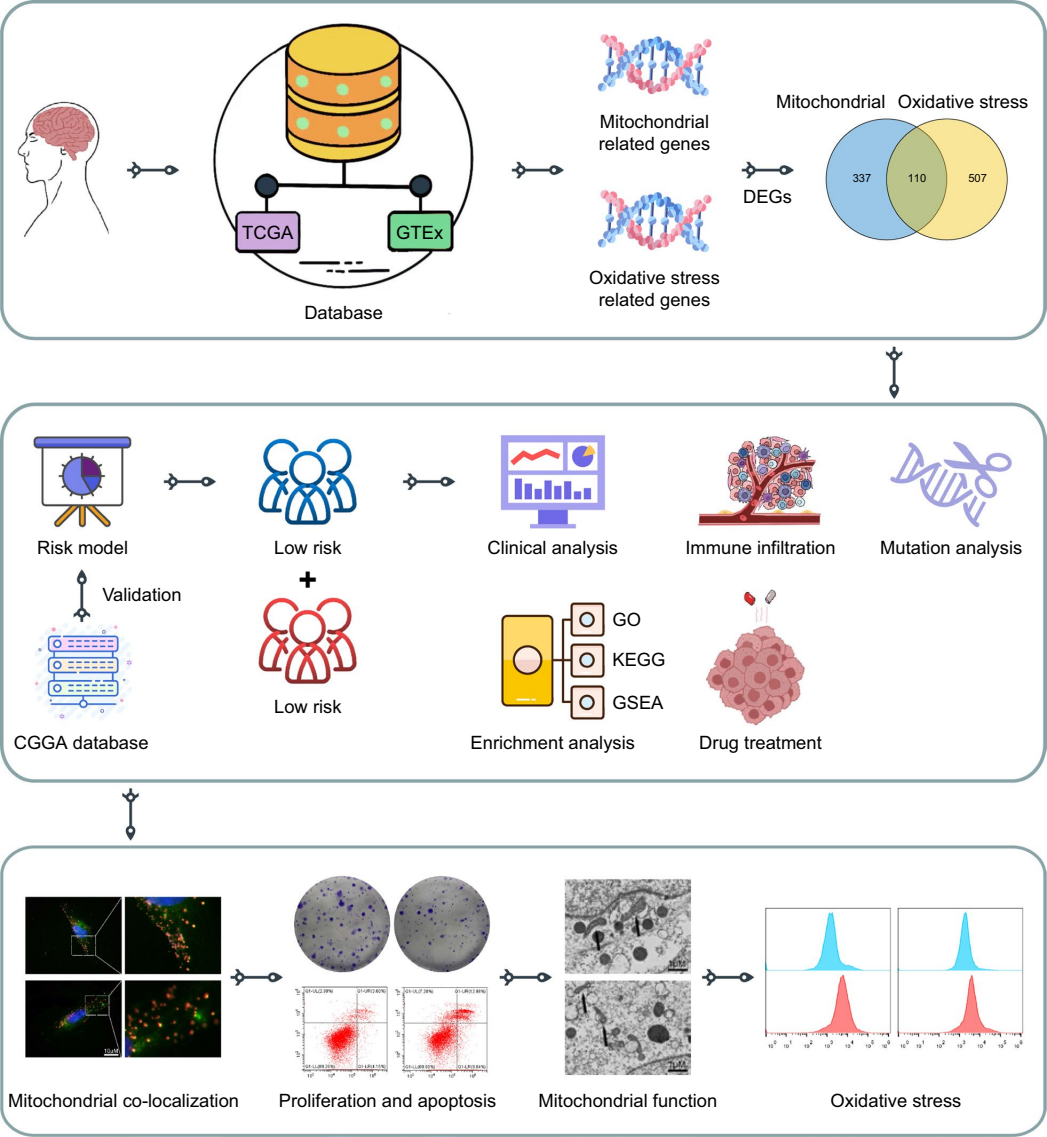
The flowchart for the study

The flowchart of this study is presented in Fig. 1. The intersection of 447 mitochondria-related DEGs and 617 oxidative stress-related DEGs identified using GBM expression data from the TCGA and GTEx databases yielded 110 DEGs. Then, the intersected genes were used for subsequent clinical, immune infiltration, mutation, enrichment analysis and follow-up experimental study.

### Identification of DEGs and construction of a risk model

**Fig. 2:**
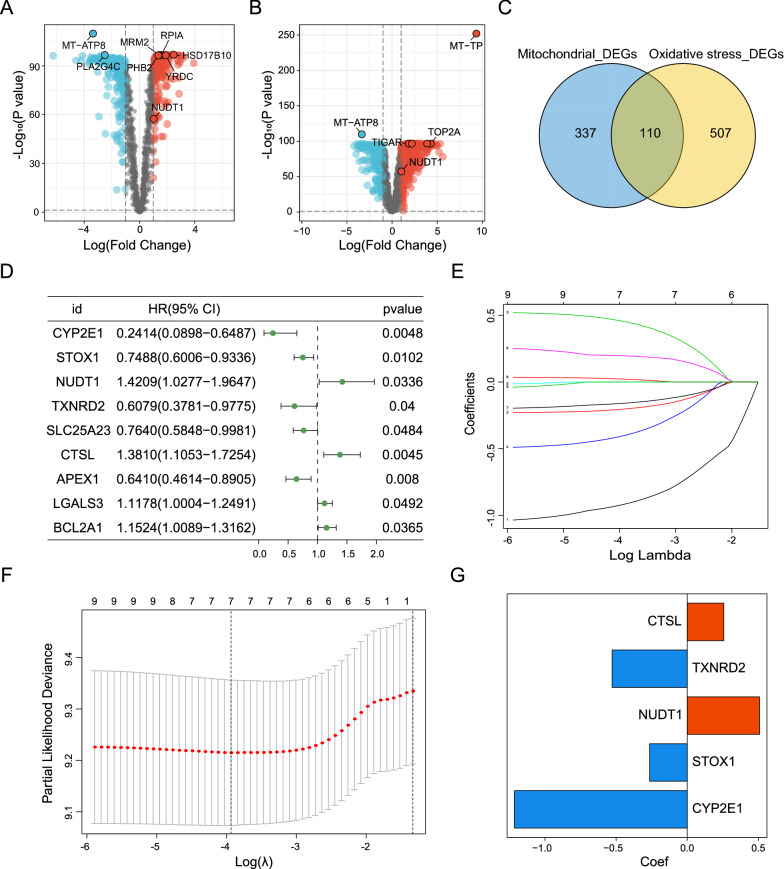
Identification of DEGs and construction of risk model. **A**, **B** Screening for mitochondria and oxidative stress-related DEGs based on the TCGA combined GTEx cohort. Red represents upregulated genes with |logFc|> 1 and P < 0.001, black indicates no significantly different genes, and blue illustrates downregulated genes with |logFC|< -1 and P < 0001. **C** Venn diagram of 110 DEGs screened in 2 DEGs cohorts. **D** Univariate cox analysis of 110 DEGs. **E**, **F** Cross-validation for optimal parameter selection in the LASSO regression model. **G** Coef values for 5 genes in the risk model

**Fig. 3:**
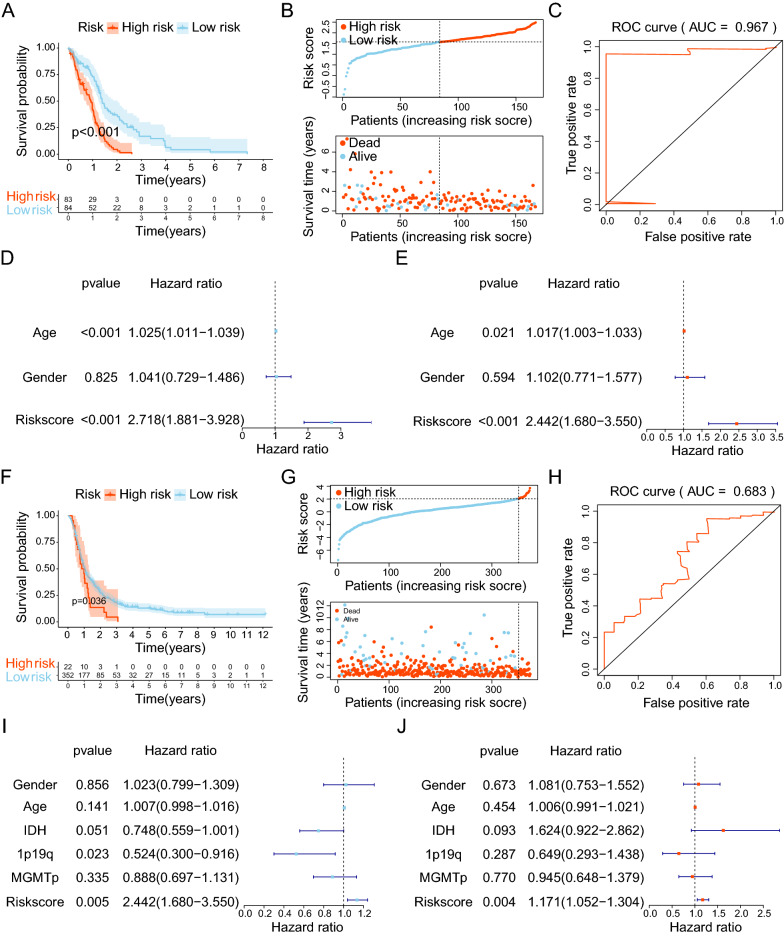
Clinical correlation analysis. **A** Kaplan–Meier survival analysis between the low- and high-risk groups in the TCGA cohort. p < 0.05 was considered significant. **B** Risk scores, survival status, and risk genes for the TCGA cohort. **C** ROC curves for the TCGA cohort risk model. **D** Univariate Cox analysis of the TCGA cohort. **E** Multivariate Cox analysis of the TCGA cohort. **F** Kaplan–Meier survival analysis between low- and high-risk groups in the CGGA validation cohort, p < 0.05 was considered significant. (**G** Risk scores, survival status, and risk genes for the CGGA cohort. **C** ROC curves for the CGGA cohort risk model. **D** Univariate Cox analysis of the CGGA cohort. **E** Multivariate Cox analysis of the CGGA cohort

To study the mRNA expression profile of GBM patients, we collected a combined cohort of TCGA and GTEx cohorts to assess the difference in gene expression level between GBM and normal samples. According to the gene expression differences of 1387 mitochondria-related genes and 2052 oxidative stress-related genes between GBM and normal tissues, 447 mitochondria-related differential genes (Fig. 2A) and 617 oxidative stress-related differential genes (Fig. 2B) were obtained, respectively. A Venn plot was generated to identify 110 intersection genes in the 2 sets (Fig. 2C). Next, univariate Cox, LASSO regression, and multivariate Cox analyses were performed for 110 genes. Univariate Cox analysis yielded 9 prognostic-related genes based on a P-value < 0.05 (Fig. 2D). LASSO regression analysis and multivariate Cox analysis identified 5 genes with predictive significance that were incorporated in the risk model (CTSL, TXNRD2, NUDT1, STOX1, and CYP2E1) (Fig. 2E–G). The risk score was calculated based on the expression levels and matching coefficients of the five genes, with the risk score = (NUDT1*0.5091)-(CYP2E1*1.2152)-(STOX1*0.2644)-(TXNRD2*0.5281) + (CTSL*0.2570). In the TCGA cohort, Kaplan–Meier analysis showed that the survival probability of GBM patients in the high-risk group was significantly shorter than in the low-risk group (Fig. 3A). The risk score-survival states-risk genes of GBM patients are shown in Fig. 3B. The ROC curve showed that the AUC value of the model based on risk score was 0.967 (Fig. 3C). During univariate and multivariate Cox analyses, the P value of risk score was less than 0.05 and the HR was more than 1, indicating that risk score was an independent risk factor for the prognosis of GBM patients (Fig. 3D, E). Finally, Kaplan–Meier survival analysis and risk score-survival status-risk genes of the CGGA verification cohort yielded similar conclusions to the analysis of the TCGA cohort (Fig. 3F, G). The AUC value of the CGGA cohort was 0.683 (Fig. 3H). Univariate and multivariate Cox analysis also showed that the risk score was an independent risk factor for GBM patients (Fig. 3I, J). Overall, our risk model exhibited a strong predictive performance.

### Overview of immune cell infiltration and tumor mutation

**Fig. 4:**
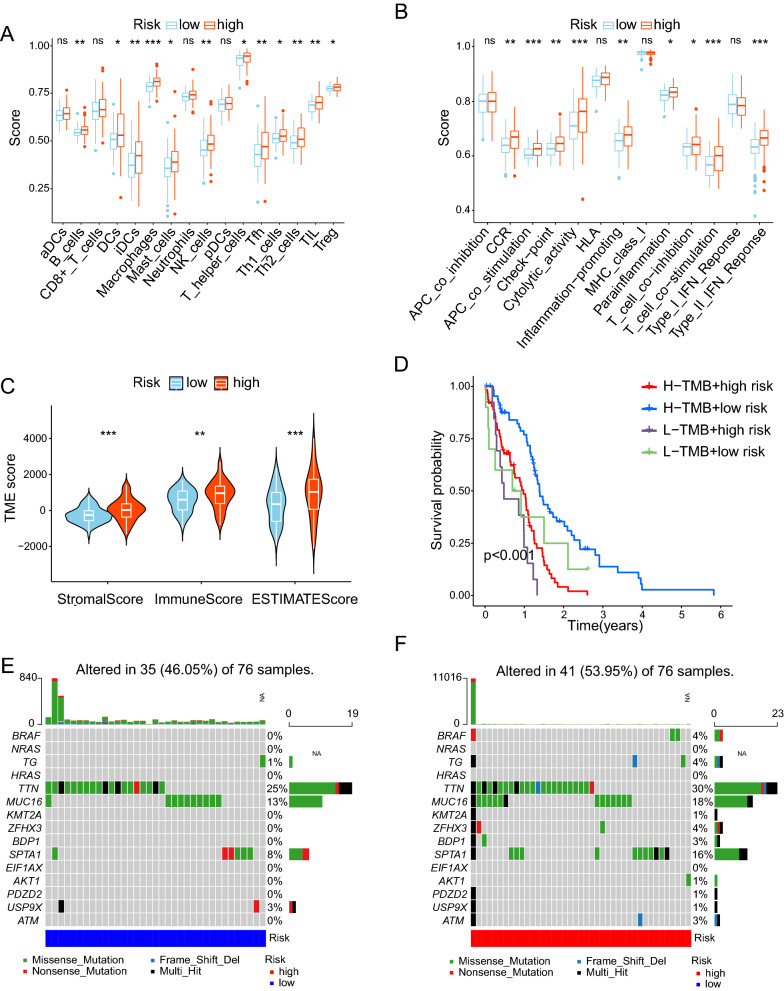
Overview of immune cell infiltration and tumor mutation. **A** Correlations between high- and low-risk groups and 16 kinds of immune cells. **B** Correlations between high- and low-risk groups with 13 kinds of immune functions. **E** Immune, Stromal, and Estimate Scores in high and low-risk groups. **D** Differences in survival probabilities between high TMB with the low-risk group, high TMB with the high-risk group, low TMB with the low-risk group, and low TMB with the high-risk group. **E** The top 15 mutated genes in the high-risk group. **F** The top 15 mutated genes in the low-risk group. ns, not statistically significant, * P < 0.05, ** P < 0.01, *** P < 0.001

We explored the correlation between immune-related cells/functions and the risk score. First, we compared the expression of 16 types of immune cells (Fig. 4A) and 13 immune cell functions (Fig. 4B) in the high- and low-risk groups using the ssGSEA algorithm. The box plots demonstrated that the level of immune cells was much higher in the high-risk group than in the low-risk group. Then, we discovered that the high-risk group had significantly higher estimate, immune, and stromal scores (P < 0.05) (Fig. 4C). During tumor mutation analysis, the patients were split into two groups into high and low tumor mutational burden (TMB) groups based on the median TMB value. Kaplan–Meier survival analysis showed that patients with high TMB had a higher survival probability than those with low TMB, and patients with low TMB and high-risk scores had the worst prognosis (Fig. 4D). We also identified the top 15 mutated genes in the high- and low-risk groups (Fig. 4E, F).

### Results of enrichment analysis

**Fig. 5:**
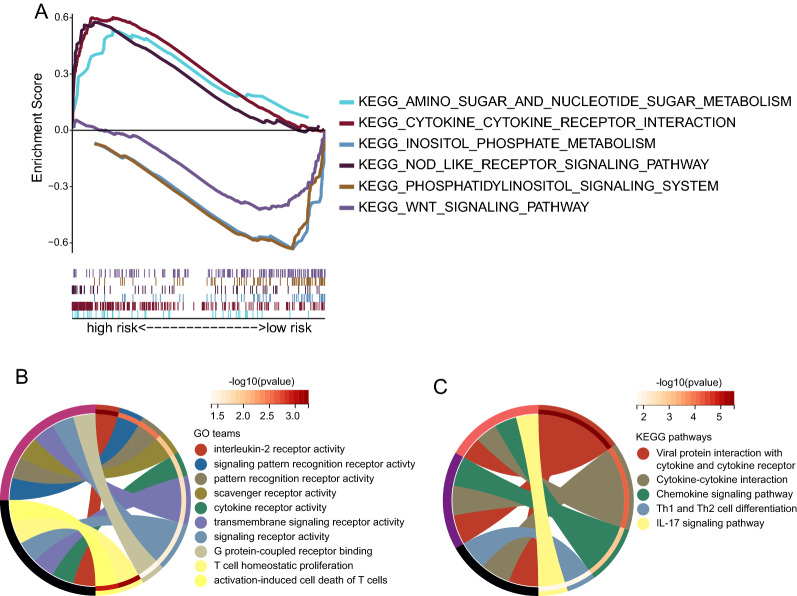
Results of enrichment analysis. **A** Enrichment analysis of GSEA in the high and low-risk groups. **B** GO enrichment analysis based on DEGs genes between high- and low-risk groups. **C** KEGG enrichment analysis based on DEGs genes between high- and low-risk groups

Functional enrichment analysis was conducted to reveal the potential biological functions and pathways associated with the risk model. According to GSEA enrichment analysis, the low-risk group was significantly enriched in inositol metabolism, whereas the high-risk group was primarily enriched in cytokine and immune-related pathways (Fig. 5A). KEGG enrichment showed that the differences between high- and low-risk groups were mainly concentrated in chemokines, and IL-17 signaling pathways (Fig. 5B). GO enrichment showed that the difference between high- and low-risk groups was mainly related to biological signal transduction and cytokines (Fig. 5C).

### Analysis of drug sensitivity

**Fig. 6:**
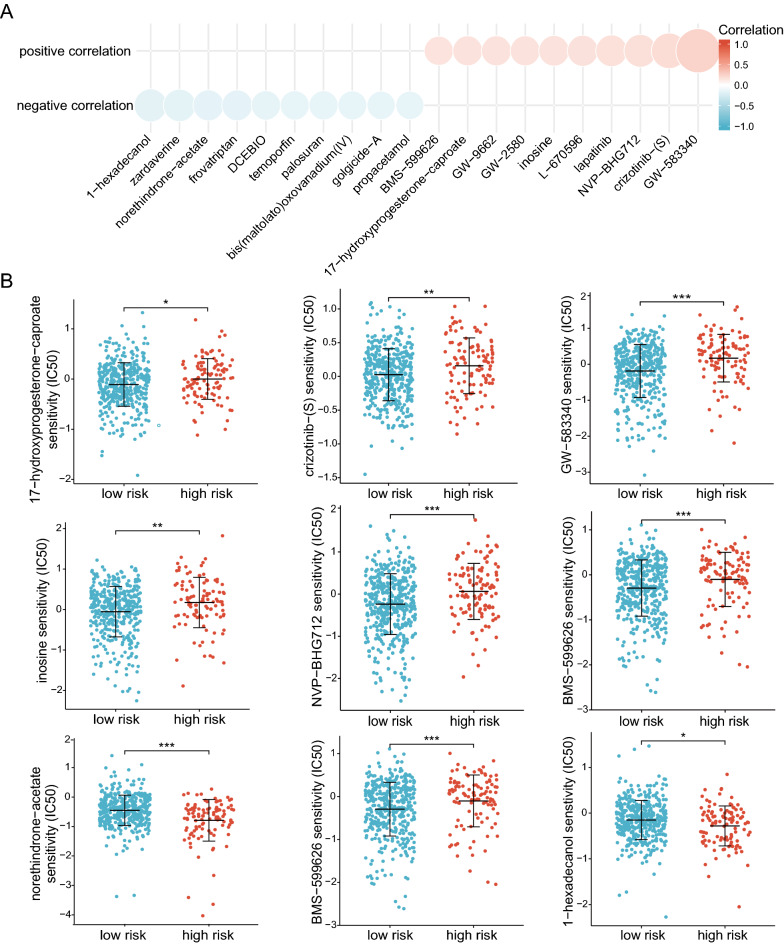
Analysis of drug sensitivity. **A** Relationship between risk score and drug sensitivities in the PRISM database. **B** Relationship between sensitivity and risk score for nine drugs. * P < 0.05, ** P < 0.01, *** P < 0.001

We examined the association between the risk score and drug sensitivity using the Prism database. 20 drugs were related to the risk score, including 1-hexadecanol, zardaverine, norethindrone-acetate, frovatriptan, DCEBIO, temoporfin, palosuran, bis(martellato)oxovanadium(IV), golgicide-A, propacetamol, BMS-599626, 17-hydroxyprogesterone-caproate, GW-9662, GW-2580, inosine, L-670596, lapatinib, NVP-BHG712, crizotinib-(S), GW-583340 (Fig. 6A). Finally, we evaluated and displayed drugs that differed considerably between the high- and low-risk groups, including 17-hydroxyprogesterone-caproate, crizotinib-(S), GW-583340, inosine, NVP-BHG712, BMS-599626, norethindrone-acetate, BMS-599626,1-hexadecanol (Fig. 6B).

### NUDT1 is elevated in GBM and colocalized in the mitochondria

**Fig. 7:**
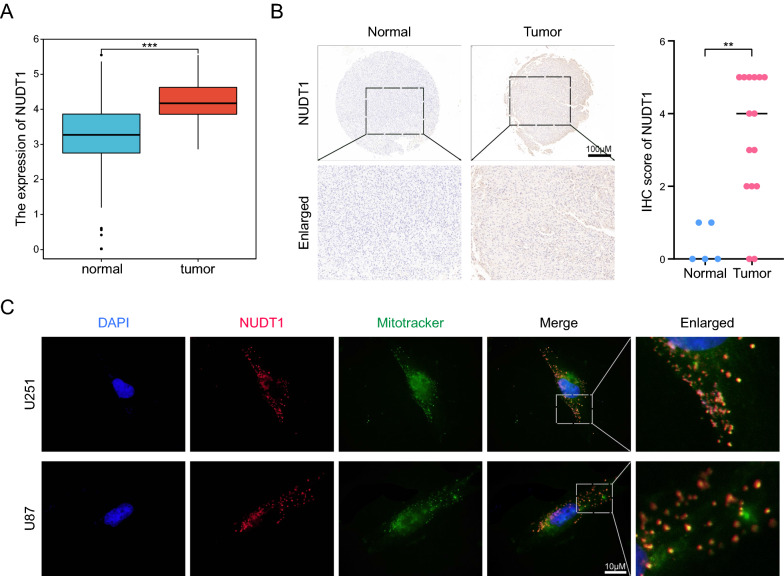
NUDT1 is elevated in GBM and co-located with mitochondria. **A** The expression of NUDT1 between the GBM and normal tissues in the TCGA cohort. **B** IHC representative images of clinical samples of NUDT1, and statistical analysis results were shown on the right. **C** Immunofluorescence assay showing the expression site of NUDT1, and the Mito-Tracker was used to label the mitochondria, scale bars: 10 μm. ** P < 0.01, *** P < 0.001

Our risk model comprised 5 candidate genes, of which NUDT1 has been proven essential in the pathogenesis of GBM [25]. However, the interaction between NUDT1 and mitochondria and oxidative stress has not been investigated in GBM. To further verify our model, NUDT1 was selected for in vitro experiments. First, the expression of NUDT1 in GBM was significantly higher than in normal tissues in the TCGA database (Fig. 7A). The same results were obtained during the IHC assay of clinical samples harvested (Fig. 7B). In addition, NUDT1 was colocalized in the mitochondria (Fig. 7C), further demonstrating the strong relationship between the two.

### Knockdown of NUDT1 induces antiproliferation and apoptosis in GBM cells

**Fig. 8:**
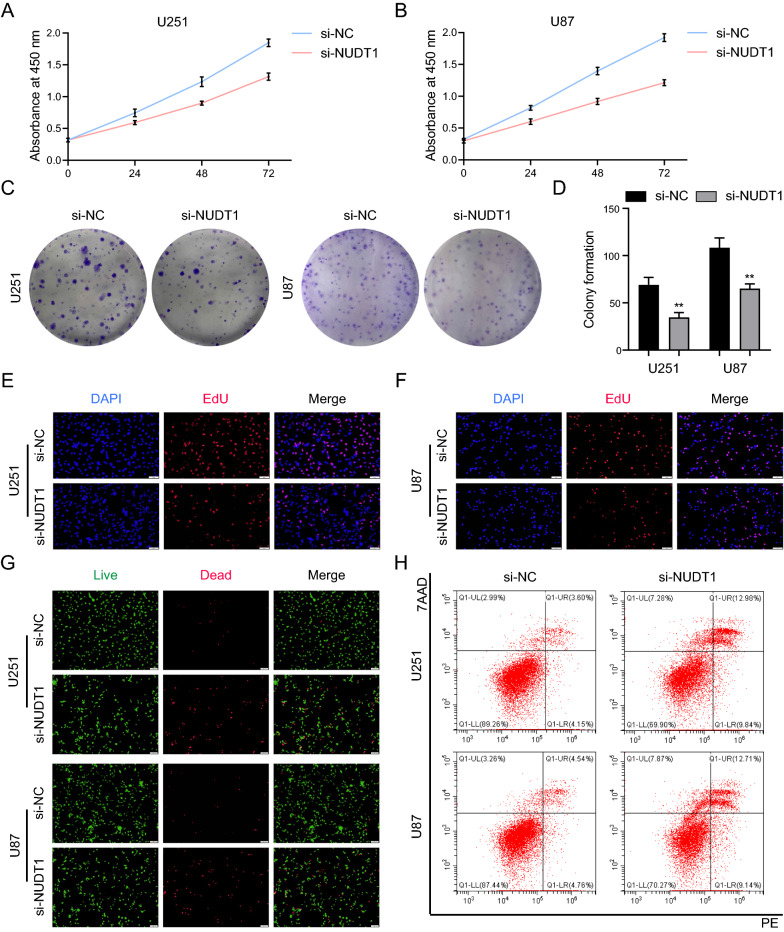
Knockdown of NUDT1 induces antiproliferation and apoptosis in GBM cells. **A** The vitality of transfected U251 cells was measured through CCK8 assay. **B** The vitality of transfected U87 cells was measured through CCK8 assay. **C**, **D** Knockdown of NUDT1 significantly reduced the quantity of clones in U251 and U87 cells. **E**–**F** The EdU assay of U251 and U87 cells after 24 h of transfection with NUDT1, scale bars: 50 μm. **G** The Calcein-AM/PI staining of U251 and U87 cells after 24 h of transfection with NUDT1, scale bars: 100 μm. **H** Knockdown of NUDT1 markedly increased the apoptosis rate of U251 and U87 cells. ** P < 0.01

**Fig. 9:**
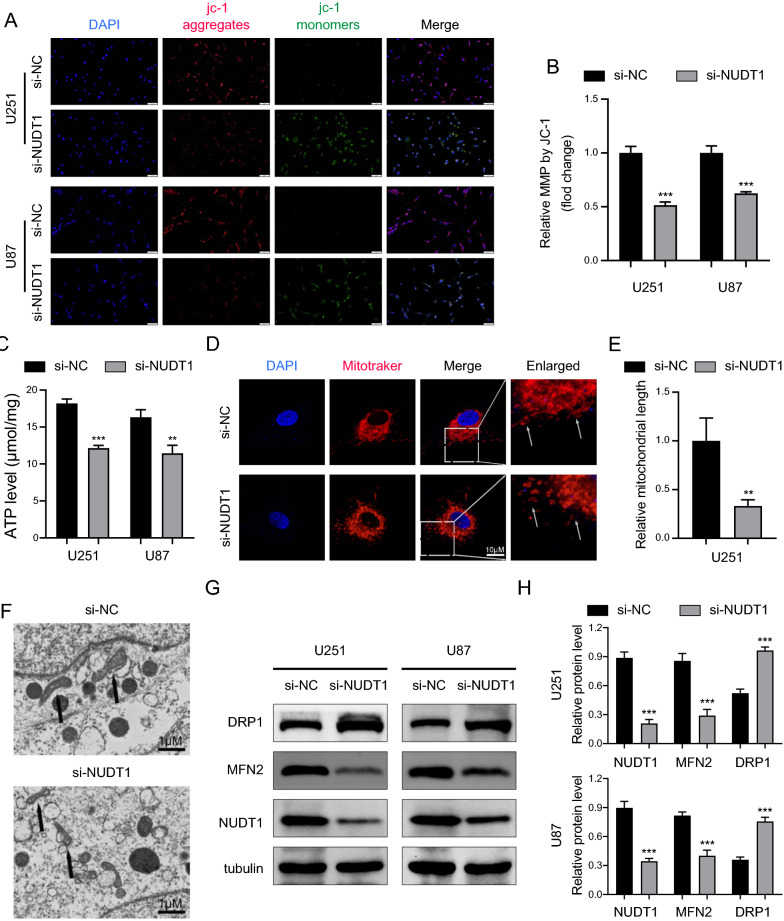
Knockdown of NUDT1 causes mitochondrial dysfunction in GBM cells. **A**, **B** JC-1 dye was used to measure the change of MMP and the results were statistically analyzed by ImageJ software, scale bars: 50 μm. **C** The ATP levels in U251 and U87 cells. **D**, **E** After 24 h of transfection, the morphology of mitochondria was observed using representative confocal images of Mitotraker, scale bars: 1 μm. **F** TEM images were used to visualize the ultrastructure of mitochondria, scale bars: 1 μm. **G** After 24 h of transfection, WB was utilized to analyze the protein expression levels of MFN2, DRP1 and NUDT1. ** P < 0.01, *** P < 0.001

We transiently transfected the NUDT1 plasmid to investigate the role of NUDT1 in GBM. WB was performed to assess the efficacy of knockdown (Fig. 9G). Subsequently, a series of experiments were carried out. First, the CCK8 assay results demonstrated that NUDT1 knockdown markedly inhibited the viability of GBM cells (Fig. 8A, B). In addition, NUDT1 knockdown drastically reduced the abundance of clone cells (Fig. 8C, D). As shown in Fig. 8E, F and Additional file 1: Fig. S1A, the quantity of EdU-positive cells in the NUDT1 knockdown group was significantly lower than in the control group. We also validated NUDT1's impact on GBM cell apoptosis. Compared to the control group, the number of dead cells in the knockdown NUDT1 group was significantly higher (Fig. 8G), and the proportion of apoptotic cells was increased (Fig. 8H and Additional file 2: Fig. S2A). Overall, our findings suggest that NUDT1 is crucial for the proliferation of GBM cells.

### Knockdown of NUDT1 causes mitochondrial dysfunction in GBM cells

Given the strong association between NUDT1 and mitochondria, we speculated whether NUDT1 regulates the proliferation and apoptosis of GBM cells by affecting mitochondria. To explore the effect of NUDT1 on mitochondrial physiological function. First, the MMP is an important indicator of mitochondrial homeostasis [26]. According to our findings, the MMP was considerably decreased when NUDT1 was knocked down (Fig. 9A, B). Mitochondria represent a major source of intracellular ATP. Compared with the control group, the NUDT1 knockdown group had lower ATP production (Fig. 9C). It is well-established that mitochondrial fragmentation is a precursor to mitochondrial dysfunction [27]. As shown in Fig. 9D, E, normal mitochondria were observed with a slender oval or rod shape, while a large number of short and spherical mitochondria were observed in the NUDT1 knockdown group. Consistently, mitochondrial atrophy and diminished or absent cristae folds were observed on TEM (Fig. 9F). It is well-known that a key component impacting mitochondrial morphology is mitochondrial dynamics, controlled by fission and fusion [28]. Furthermore, WB was used to detect mitochondrial morphogenetic proteins. Knockdown of NUDT1 increased the expression of the mitochondrial fission protein DRP1 while inhibiting the expression of the fusion protein Mitofusin 2 (MFN2) (Fig. 9G). In summary, our findings indicated that NUDT1 is essential for maintaining mitochondrial homeostasis and dynamics.

### Knockdown of NUDT1 induces oxidative stress in GBM cells

**Fig. 10:**
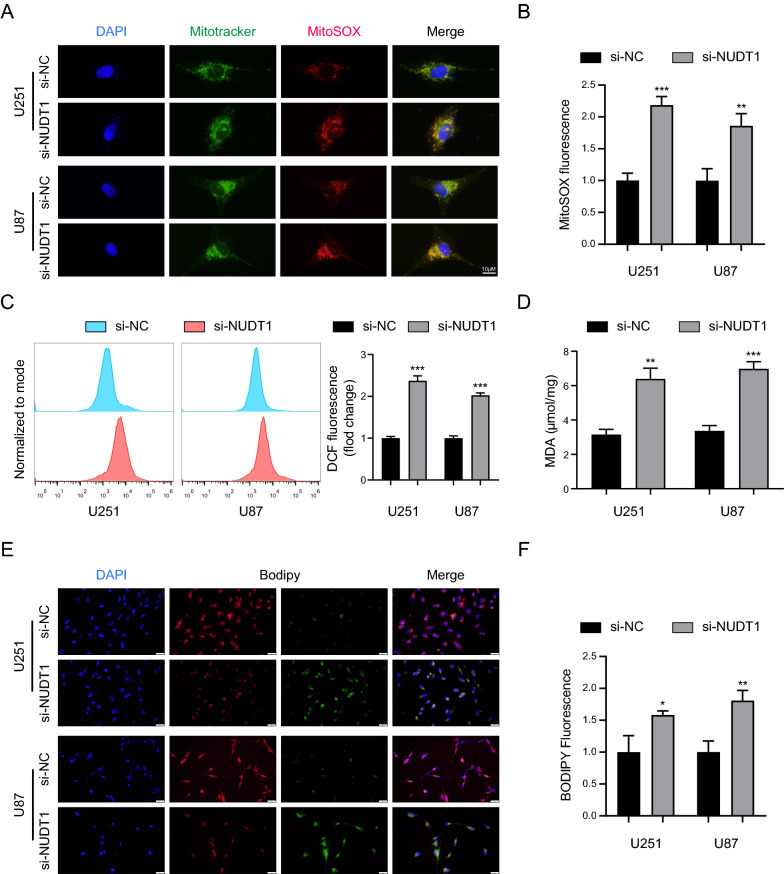
Knockdown of NUDT1 induces oxidative stress in GBM cells. **A**, **B** The fluorescent images revealed the changes in the mitochondrial ROS of U251 and U87 cells, scale bars: 10 μm. **C** The total ROS was detected in U251 and U87 cells after 24 h of transfection with NUDT1. **D** The MDA level in U251 and U87 cells. **E**, **F** The BODIPY was utilized to measure the level of lipid peroxidation in U251 and U87 cells, scale bars: 20 μm. * P < 0.05, ** P < 0.01, *** P < 0.001

The mitochondria represent the primary source of intracellular ROS, and dysfunctional mitochondria can result in greater levels of ROS, which can further harm the mitochondria. This cascade response ultimately results in cell death, demonstrating the tight correlation between mitochondria and oxidative stress [29]. To investigate NUDT1's impact on oxidative stress in GBM cells, we measured the levels of ROS produced by the mitochondria, and the fluorescence results revealed that NUDT1 knockdown dramatically increased mitochondrial ROS production (Fig. 10A, B). Furthermore, total ROS levels in GBM cells were increased (Fig. 10C). In addition, NUDT1 knockdown resulted in a significant rise in MDA levels and lipid oxidation in GBM cells (Fig. 10D–F). Overall, our findings implied that NUDT1 knockdown induces mitochondrial oxidative damage in GBM cells.

## Discussion

GBM is a highly invasive malignant tumor with 2-year and 5-year survival rates of 21.3% and 13.8%, respectively, and there has been no significant improvement in the past decade [30]. Notwithstanding that the past decade has witnessed unprecedented medical progress, the current standard of care for GBM remains maximum safe surgical resection combined with postoperative radiation and chemotherapy [31]. Due to the prevalence of drug resistance, the first-line chemotherapy drug TMZ is increasingly ineffective and practically all GBM patients experience relapses [32]. Combined with the high aggressiveness and lethality of GBM, the prognosis of GBM patients is extremely poor [33]. Therefore, there is an urgent need for a novel and effective treatment to address these challenges. Bioinformatics represents a comprehensive and systematic new technology that has received significant attention with the advent of precision medicine. Additionally, the advancement of sequencing technology and the updating of databases have improved the accuracy and dependability of bioinformation technology.

It is widely acknowledged that the mitochondria consume oxygen and generate energy through the tricarboxylic acid cycle and oxidative phosphorylation, which is an important driving force for cells [34]. Mitochondria are dynamic organelles. The stability of their dynamics and morphology is essential to maintain some physiological functions, including signal transduction, proliferation, transfer, oxidative stress, and death [35]. Over the past few years, new treatment approaches have been developed, focusing on oxidative stress caused by mitochondrial damage. Wu Y et al. analyzed the predictive role of mitochondrial dysfunction and oxidative stress pathway related genes in clear cell renal cell carcinoma and described the physiological function and clinical value of these genes [36]. Luo W et al. developed a risk scoring model based on 4 mitochondrial related genes to predict the prognosis of breast cancer patients [37]. However, the role of mitochondria and oxidative stress in GBM pathogenesis has been largely understudied compared to other tumors.

Here, we screened and identified mitochondria and oxidative stress related core genes through bioinformatics and constructed a prognosis model. First, we collected mitochondrial and oxidative stress related genes from various public databases. Then, the differential expression analysis, LASSO regression and multivariate Cox analyses were used to construct and verify the risk score characteristics based on 5 oxidative stress related mitochondrial genes in GBM. Our model exhibited a superior predictive performance than the traditional prognosis model of apoptosis, autophagy, epithelial-mesenchymal transition (EMT), and other associated genes [38–40]. Immunotherapy has become the fourth most popular treatment after surgery, radiotherapy and chemotherapy [41]. Interestingly, our risk model was closely related to tumor immunity and mutation and suggested that immunotherapy might be more effective for GBM patients with high-risk scores. Then, functional enrichment analysis revealed that our model was mostly associated with immunology, energy metabolism, and cytokines. The results of immunology and cytokines overlapped with immunotherapy analysis, further indicating that our prognosis model was inseparable from tumor immunity. Mitochondria is the main site of energy metabolism, and tumor cells may obtain resistance to stress signals, and metabolic reprogramming to meet the needs of energy [42]. Therefore, targeting energy metabolism of cancer cells has been considered as a promising strategy. Finally, we identified several drugs related to the risk score. Some studies reported that GW-583340 increased ROS and induced breast cancer cell death [43]. 1-hexadecanol was used to synthesize temozolomide ester compounds (TMZEs) to overcome the drug resistance of glioma cells [44]. These provided us with potential therapeutic drugs. In conclusion, our model provided an effective basis for diagnosing and treating GBM patients, and the 5 core genes that make up the model have been validated to be strongly related to tumor progression and patient prognosis. In this respect, Dong Q et al. discovered that reducing the expression of Cathepsin L (CTSL) prevented GBM cells from proliferating and migrating while also inducing mitochondrial-related apoptosis [45]. Mitochondrial thioredoxin reductase (TXNRD2) is highly expressed in numerous malignancies and is associated with poor prognosis and treatment resistance [46]. Knockdown of TXNRD2 could induce oxidative stress and trigger the apoptotic pathway [47]. Storkhead box 1 (STOX1) is an independent prognostic factor of glioma patients [48]. In the placenta, STOX1 can reportedly affect the balance of nitroso redox by regulating mitochondrial function [49]. Ye L et al. demonstrated that the down-regulation of cytochrome P450 family 2 subfamily E member 1 (CYP2E1) predicted a poor prognosis of glioma patients [50], and increased lipid peroxidation and generated oxidative stress [51]. Nucleoside diphosphate linked moiety X-type motif 1 (NUDT1), also known as MTH1, encoded 8-oxo-2′-deoxyguanosine triphosphatase (8-oxo-dGTPase), has been reported to hydrolyze 8-oxo-dGMP to prevent the buildup of oxides in mitochondria and nuclei [52]. Moreover, the inhibitor of NUDT1 yielded an anti-cancer effect in gastric cancer by decreasing mitochondrial function [53].

The interaction between NUDT1, mitochondria, and oxidative stress in GBM has not been precisely studied. As a result, we selected NUDT1 for in vitro experiments to confirm its impact on the physiological functions of GBM. Finally, the results of public database and clinical samples showed that NUDT1 was highly expressed in GBM. Moreover, the expression of NUDT1 was concentrated in mitochondria, which also indicated that its function was closely related to mitochondria. Subsequently, we found that knockdown of NUDT1 inhibited the proliferation and induced apoptosis of GBM cells. Furthermore, the results showed that knockdown of NUDT1 damaged mitochondrial function and induced oxidative stress. Therefore, we speculated that NUDT1 might induce GBM cell apoptosis through mitochondrial pathway. In short, our findings validated the reliability of our model by demonstrating the importance of NUDT1 in maintaining the development, proliferation, mitochondrial function, and redox balance of GBM cells.

Despite the thorough planning that went into our research, there were still certain constraints. Our findings obtained by bioinformatics analysis were validated by in vitro experiments. Nonetheless, additional in-depth mechanism research and animal experiments are needed. Additionally, the prognostic model developed was based on retrospective public data. Accordingly, prospective research and clinical case analysis are required to validate our findings.

## Conclusion

This is the first study to comprehensively and systematically analyze mitochondria and oxidative stress-related genes in GBM, and construct a model with good prediction performance for clinical prognosis, immune response and targeted therapy of GBM patients. In addition, further in vitro experiments demonstrated that NUDT1 significantly regulated the proliferation, apoptosis, mitochondrial homeostasis, and oxidative stress of GBM cells. Overall, our study provides novel insights into the mechanism of GBM and potential treatment strategies for this patient population.

## Supplementary Information

**Additional file 1: Figure S1.** The statistical analysis of the EdU assay in U251 and U87 cells.

**Additional file 2: Figure S2.** The statistical analysis of flow cytometry in U251 and U87 cells.

**Additional file 3: Table S1.** The basic information of enrolled patients.

## Data Availability

The original datas presented in the study are included in the article. Further inquiries can be directed to the corresponding author.
