# Supplementary figures and images for: Identification and validation of a novel prognostic signature based on mitochondria and oxidative stress related genes for glioblastoma
Source: J Transl Med. 2023 Feb 22;21:136. doi: 10.1186/s12967-023-03970-6 (PMC9948483; doi:10.1186/s12967-023-03970-6)

A

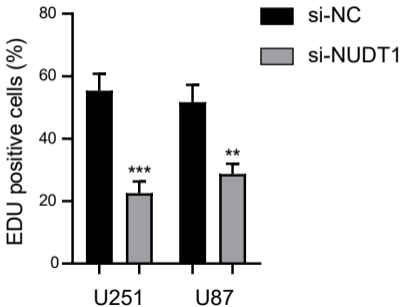

Supplement: Supplementary file 1 — Additional file 1: Figure S1. The statistical analysis of the EdU assay in U251 and U87 cells. [file 12967_2023_3970_MOESM1_ESM.pdf]

A

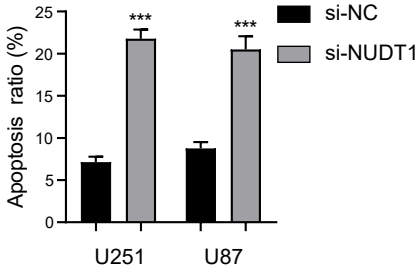

Supplement: Supplementary file 2 — Additional file 2: Figure S2. The statistical analysis of flow cytometry in U251 and U87 cells. [file 12967_2023_3970_MOESM2_ESM.pdf]
