# Supplementary material for: Identification and validation of a novel prognostic signature based on mitochondria and oxidative stress related genes for glioblastoma
Source: J Transl Med. 2023 Feb 22;21:136. doi: 10.1186/s12967-023-03970-6 (PMC9948483; doi:10.1186/s12967-023-03970-6)
Supplement: Supplementary file 3 — Additional file 3: Table S1. The basic information of enrolled patients. [file 12967_2023_3970_MOESM3_ESM.pdf]

Characteristic	TCGA-GBM(n=167)	CGGA-GBM(n=374)
**Survival status**		
Alive	30 (5.5%)	53 (9.8%)
Dead	137 (25.3%)	321 (59.3%)
**Age, median (IQR)**	60 (50.5, 69)	49 (40, 58)
**Gender**		
Female	59 (10.9%)	148 (27.4%)
Male	108 (20%)	226 (41.8%)
**IDH_mutation_status**		
Mutant	0 (0%)	84 (23.1%)
Wildtype	0 (0%)	280 (76.9%)
**1p19q_codeletion_status**		
Codel	0 (0%)	19 (5.5%)
Non-codel	0 (0%)	324 (94.5%)
**MGMTp_methylation_status**		
methylated	0 (0%)	169 (51.5%)
un-methylated	0 (0%)	159 (48.5%)
